# High Intra-Tumor Transforming Growth Factor Beta 2 Level as a Predictor of Poor Treatment Outcomes in Pediatric Diffuse Intrinsic Pontine Glioma

**DOI:** 10.3390/cancers15061676

**Published:** 2023-03-09

**Authors:** Fatih M. Uckun, Sanjive Qazi, Vuong Trieu

**Affiliations:** 1 Ares Pharmaceuticals, Immuno-Oncology Program, St. Paul, MN 55110, USA; 2 Oncotelic Therapeutics, 29397 Agoura Road, Suite 107, Agoura Hills, CA 91301, USA

**Keywords:** DIPG, GBM, glioma, TGF beta, TGFB2, RNAseq, mRNA

## Abstract

**Simple Summary:**

Pediatric diffuse intrinsic pontine gliomas (DIPGs) are one of the most aggressive and deadliest childhood brain tumors. The purpose of the present study was to evaluate the clinical significance of amplified expression levels of transforming growth factor beta 2 (TGFB2) in the tumor tissue specimens from DIPG patients. Our findings provide the first evidence that high level TGFB2 expression is associated with a poor treatment outcome in DIPG. The reported results also support the notion that further evaluation of the clinical potential of new strategies targeting TGFB2 in pediatric DIPG is warranted.

**Abstract:**

Here, we report that tumor samples from newly diagnosed pediatric diffuse intrinsic pontine glioma (DIPG) patients express significantly higher levels of transforming growth factor beta 2 (TGFB2) messenger ribonucleic acid (mRNA) than control pons samples, which correlated with augmented expression of transcription factors that upregulate TGFB2 gene expression. Our study also demonstrated that RNA sequencing (RNAseq)-based high TGFB2 mRNA level is an indicator of poor prognosis for DIPG patients, but not for pediatric glioblastoma (GBM) patients or pediatric diffuse midline glioma (DMG) patients with tumor locations outside of the pons/brainstem. Notably, DIPG patients with high levels of TGFB2 mRNA expression in their tumor samples had significantly worse overall survival (OS) and progression-free survival (PFS). By comparison, high levels of transforming growth factor beta 3 (TGFB3) mRNA expression in tumor samples was associated with significantly better survival outcomes of DIPG patients, whereas high levels of transforming growth factor beta 1 (TGFB1) expression was not prognostic. Our study fills a significant gap in our understanding of the clinical significance of high TGFB2 expression in pediatric high-grade gliomas.

## 1. Introduction

Pediatric diffuse intrinsic pontine gliomas (DIPGs) belong to the most aggressive and deadliest childhood brain tumors classified as histone H3 Lysine 27 (H3K27)-altered diffuse midline gliomas (DMG), including the H3 K27-mutant as well as H3 wildtype subtypes with overexpression of the EZH inhibitory protein (EZHIP) [1,2,3]. Complete resection of DIPGs, which are the second most common malignant pediatric brain tumors, is not possible due to its anatomic location in the brainstem and its rapid infiltrative growth. DIPG is associated with a poor overall survival (OS) despite contemporary radiation therapy/radio-chemotherapy strategies, and it is one of the driving contributors to cancer-related mortality in children (median OS < 1 year) [4,5,6,7,8,9,10]. In spite of numerous clinical trials of chemotherapeutic agents, immune-oncology drugs, and specific targeted therapies aimed at improving the survival outcome of pediatric patients with DIPG, little progress has been achieved and the prognosis of DIPG remains dismal, with a median survival time of approximately 10 months, and a two-year survival rate of less than 10 percent [4,5,6,7,8,9,10]. Furthermore, there is no standard treatment for progressive DIPG after the failure of frontline radiation therapy, and no salvage regimen has been shown to extend the OS [11]. Effective treatment strategies that could potentially improve the dismal prognosis of these children are urgently needed and their discovery represents a main focus of translational and clinical research in contemporary neuro-oncology [12,13,14,15,16,17,18,19,20,21,22,23,24,25,26,27,28,29]. Several interventional strategies are being explored, such as immunotherapy with immune-checkpoint inhibitors or T cells with a chimeric antigen receptor (CAR) (“CAR-T cells”), inhibition of signal transduction pathways with small molecule drugs, and biotherapy with fusion toxins administered via convection-enhanced delivery (CED) or oncolytic viruses [12,13,14,15,16,17,18,19,20,21,22,23,24,25,26,27,28,29].

Transforming growth factor-beta (TGFB) is a disulfide-linked, homodimeric cytokine with a pleiotropic activity profile that has been implicated in oncogenesis as well as suppression of host anti-tumor immunity within the tumor microenvironment (TME). Augmented transforming growth factor beta (TGFB) signaling pathway activity mediated by autocrine or tumor-associated macrophages (TAM)-derived overproduction has been implicated in the aggressive biology and poor overall survival of adult high-grade glioma patients by promoting invasive and rapid glioma cell growth [30,31,32,33,34,35]. The TGFB pathway has also been shown to contribute to a “cold” TME in high-grade gliomas via its immunosuppressive effects characterized by inhibition of CD8-antigen-positive cytotoxic T cells, natural killer (NK) cells, and activation of regulatory T cells (Tregs) as well as myeloid derived suppressor cells (MDSCs) [30,31,32,33,34,35,36]. The TGFB pathway has emerged as a possible therapeutic target for high-grade gliomas. The FDA-approved TGFB inhibitor Pirfenidone (5-methyl-1-phenyl-2(1 H)-pyridone, PFD) has been shown to inhibit TGFB expression in malignant glioma cells [37]. A synthetic TGFB2 mRNA-targeting anti-sense phosphorothioate oligodeoxynucleotide (S-ODN) exhibited promising single-agent clinical activity associated with durable complete and partial responses in adult patients with recurrent or refractory glioblastoma and anaplastic astrocytoma when administered intratumorally via CED [37,38]. In a recent study that employed a microarray-based gene expression platform, we found that TGFB2 mRNA levels were selectively amplified in primary tumor samples from 29 pediatric DIPG patients compared to normal samples and primary tumor samples from low-grade glioma patients [39]. Although these results prompted the hypothesis that TGFB2 mRNA could serve as a molecular target for the treatment of pediatric DIPG [40], the clinical significance of these observations remains unknown.

The purpose of the present study was to evaluate the clinical prognostic significance of high tumor TGFB2 mRNA levels in pediatric DIPG, as measured by RNAseq. We hypothesized that pediatric DIPG patients who have amplified TGFB2 mRNA expression in their brain tumor tissues would exhibit a more aggressive disease with a worse prognosis. Here, we demonstrate that newly diagnosed pediatric DIPG patients with augmented TGFB2 mRNA levels in their primary tumor samples, but not pediatric DIPG patients with augmented transforming growth factor beta 1 (TGFB1) or transforming growth factor beta 3 (TGFB3) mRNA levels, have significantly worse progression-free survival (PFS) as well as overall survival (OS) than other pediatric DIPG patients. High TGFB2 mRNA level was a poor prognostic indicator for DIPG patients but not for DMG patients whose tumors were outside of the pons/brainstem in locations such as the cerebellum and thalamus or pediatric glioblastoma (GBM) patients.

## 2. Materials and Methods

We downloaded clinical metadata and RNA sequencing (RNAseq)-based mRNA expression data for 41 pediatric DMG patients (mean age at diagnosis in months = 7.02 ± 0.44; median = 6; range = 2–14) and 116 pediatric GBM patients (mean age at diagnosis in months = 60.01 ± 1.24; median = 60.4; range = 21–89.3) regarding TGFB isoforms TGFB1, TGFB2, and TGFB3 from the genomic data set repository stored in the cBioPortal for Cancer Genomics (https://pedcbioportal.kidsfirstdrc.org/ (accessed on 4 November 2022)) using the interactive web interface with full filtering functionality provided by the portal [41,42]. By utilizing the cBioPortal database and an archived dataset that was compiled from harmonized and annotated across multiple data consortiums, such as the open Pediatric Brain Tumor Atlas (PBTA) Project (Project ID = openpbta) and the Pacific Pediatric Neuro-Oncology Consortium Clinical Genomics Atlas (Project ID = pbta_pnoc) [10,43,44,45], we examined the effect of TGFB2 mRNA expression levels on PFS and OS outcomes. The general treatment strategies are outlined in the clinical trials associated with the treatment of DIPG patients (https://clinicaltrials.gov/ct2/show/NCT02274987 (accessed on 1 January 2023)) that included standard radiation therapy followed by biomarker-guided specialized therapy with FDA-approved targeted therapeutics drugs, which was guided by gene expression analysis, whole exome-sequencing (WES), and predictive modeling. As a limitation of this study, patient-specific treatment information was not available through the cBioPortal database.

The downloaded mRNA expression levels of TGFB1, TGFB2, TGFB3, Transforming Growth Factor Beta Receptor 1 (TGFBR1), Transforming Growth Factor Beta Receptor 2 (TGFBR2) and Transforming Growth Factor Beta Receptor 3 (TGFBR3) were reported using the RNAseq V2 values (datafiles for the expression profiles appended with “mRNA_expression_(RNA_Seq_V2_RSEM).txt”) normalized to “transcripts per million” (“TPM”) values calculated using RSEM, which is a software package for estimating gene and isoform expression levels from RNAseq data [46]. The RSEM-based process consists of two main steps: 1. A set of reference transcript sequences are generated for gene level mRNA abundance assessment; 2. A set of RNAseq reads are aligned to these reference transcripts for TPM abundance estimations. This process allows for a direct comparison of mRNA abundance and ranking across samples.

The Kaplan–Meier (KM) method, log-rank chi-square test, and the software packages survival_3.2-13, survminer_0.4.9 and survMisc_0.5.5 operated in the R environment were used to compare the PFS and OS outcomes of patient subsets. Graphical representations of the treatment outcomes were generated using graph drawing packages implemented in the R programming environment: dplyr_1.0.7, ggplot2_3.3.5, and ggthemes_4.2.4. The statistical significance of differences in the outcomes of the compared patient subsets was examined using the log-rank chi-square test and *p*-values less than 0.05 were deemed significant.

TBFB1, TGFB2, and TGFB3 mRNA expression values for normal pons specimens measured by RNAseq (rna_tissue_hpa.tsv.zip) were downloaded from https://www.proteinatlas.org/about/download accessed on 19 December 2022. We compiled the mRNA expression values (in TPM) for 29 distinct pons regions of the brain by filtering the “Tissue Group” annotations the accompanying description file (“rna_tissue_hpa_description.tsv.zip”). A two-way analysis of variance (ANOVA) model was used to compare the TGFB1/TGFB2/TGFB3 mRNA expression levels for the normal pons specimens with the TGFB1/TGFB2/TGFB3 mRNA expression levels in brain tumor specimens from 41 DIPG patients. Contrasts were performed between normal pons and DIPG samples for each of the transcripts and *p*-values were false discovery rate (FDR)-adjusted. Calculations were performed using multcomp_1.4-17 and emmeans_1.7.0 statistical packages in R version 4.1.2 (1 November 2021) with RStudio front end (RStudio 2021.09.0 + 351 “Ghost Orchid” Release)). Bar chart graphics were constructed using the ggplot2_3.3.5 R package.

mRNA expression levels of TGFB2 in log_2_ TPM were correlated with the mRNA expression levels for 11 transcription factors known to augment TGFB2 expression, namely activating transcription factor 1 (ATF1), activating transcription factor 2 (ATF2), cyclic AMP-responsive element binding protein 1 (CREB1), E1A binding protein P300 (EP300), forkhead box protein O3 (FOXO3), polymerase II subunit A (POLR2A), regulatory factor X1 (RFX1), specificity protein 1 transcription factor (SP1), TATA-box-binding protein (TBP), upstream transcription factor 1 (USF1), and upstream transcription factor 2 (USF2) across 41 DIPG patients. Pairwise correlation coefficients were determined for all transcript combinations and visualized on a heatmap which was color-coded from positive correlations (red = +1) to negative correlations (blue = −1). The clustering algorithm identified the co-regulated sets of genes using the statistical package ggcorrplot_0.1.3 implemented in R. T-test was used to test the null hypothesis that the Pearson correlation coefficient was equal to zero. Significant correlations were identified for *p*-values less than 0.05 and FDR less than 0.10.

Normalized archived transcriptome profiling datasets acquired from the Gene Expression Omnibus web portal (https://www.ncbi.nlm.nih.gov/geo/ accessed 23 November 2021), including raw CEL files obtained using the Human Genome U133 Plus 2.0 Array platform for DIPG (N = 29; GSE26576), normal control samples (N = 2; GSE26576), and pediatric GBM patients harboring H3K27M mutations (N = 5, GSE34824; N = 7, GSE49822) [39] were also utilized as an independent validation dataset to compare the mRNA expression levels of TGFB1, TGFB2, and TGFB3 in normal control samples vs. brain tumor specimens from 41 patients with pediatric DIPG or H3K27M-mutant pediatric GBM. The normalization procedure to determine log_2_-transformed mRNA expression levels employed the method of Robust Multi-array Averaging (RMA), as previously described [39]. mRNA expression levels were calculated using Aroma Affymetrix statistical packages (aroma.affymetrix_3.2.0, aroma.core_3.2.2 and aroma.light_3.24.0) run in the RStudio environment (R version 4.1.2 (1 November 2021), RStudio 2021.09.0 Build 351). Statistical comparisons were performed using an ANOVA statistical model. FDR-adjusted *p*-values less than 0.05 were deemed significant. mRNA expression levels in DIPG/H3K27M-mutant GBM patients for TGFB1, TGFB2, and TGFB3 were visualized using heatmaps, as described [39]. Expression levels of TGFB2 mRNA (log_2_ RMA) were correlated to mRNA a two-way ANOVA model: expression levels for 11 transcription factor genes known to augment TGFB2 expression; ATF1, ATF2, CREB1, EP300, FOXO3, POLR2A, RFX1, SP1, TBP, USF1, and USF2.

## 3. Results

### 3.1. Tumor Specimens from Pediatric Patients with DIPG Contain Higher Levels of TGFB2 mRNA but Not TGFB1 mRNA or TGFB3 mRNA, Compared to Normal Pons Specimens

**Figure 1:**
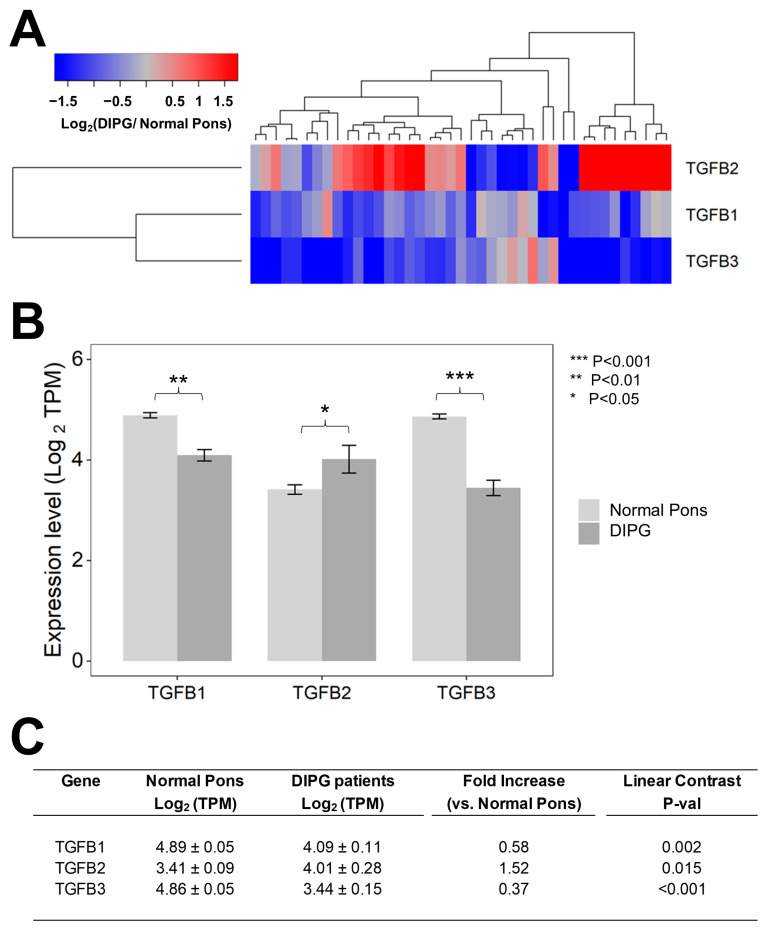
Selective upregulation of TGFB2 mRNA expression in DIPG tumor samples. Archived TGFB1/2/3 mRNA expression levels (in log_2_-transformed TPM) for primary tumor samples from 41 DIPG patients, including 11 DIPG patients with unknown H3K27M mutational status, 4 H3K27M- mutant DIPG patients, and 26 H3K27M-mutant DMG patients, whose brain tumors were localized to pons/brainstem, obtained from cBioPortal were compared to TGFB1/2/3 mRNA expression levels (log_2_ TPM) in normal pons samples from 29 pons regions (downloaded from https://www.proteinatlas.org/about/download, accessed on 17 December 2022). The TGFB2 expression levels for these 29 normal pons regions were determined by averaging the TPM values for 2–8 independent samples/region from 21 subjects. Expression of log_2_-transformed TPM values for TGFB1/2/3 mRNA levels in the tumor samples from DIPG patients were mean-centered to the mRNA expression levels in normal pons samples and depicted in the heatmap (**A**) representing overexpression (red color) or underexpression (blue color). (**B**) The bar charts illustrate reduced expression levels for TGFB1 and TGFB3 mRNA but amplified expression levels for TGFB2 mRNA, in tumor specimens from DIPG patients (dark grey bars) when compared to normal pons samples (light grey bars). (**C**) Statistical significance of differences in TGFB1/2/3 mRNA expression levels (in log_2_-transformed TPM values) was assessed using two-way ANOVA with linear contrasts using FDR-adjusted *p*-values. We documented a statistically significant 1.52-fold increase in TGFB2 mRNA levels (*p* = 0.015) along with statistically significant decreases in TGFB1 mRNA (1.72-fold decrease; *p* = 0.002) and TGFB3 (2.7-fold decrease; *p* < 0.001) mRNA levels.

We first set out to compare the RNAseq-based mRNA levels for TGFB1/2/3 isoforms in 41 primary DIPG samples vs. 29 normal pons specimens (Figure 1). Notably, the average (mean ± SE) TGFB2 mRNA level in primary DIPG samples was 1.5-fold higher than the average TGFB2 mRNA level in normal pons specimens (4 ± 0.3 vs. 3.4 ± 0.1, *p* = 0.015) (Figure 1). By contrast, both TGFB1 and TGFB3 mRNA levels in DIPG samples were significantly lower (1.7-fold lower TGFB1 mRNA level, *p* = 0.0002 and 2.7-fold lower TGFB3 mRNA level, *p* < 0.001) than the corresponding levels in normal pons samples, as reflected by the blue color in the heat map of the hierarchical cluster (Figure 1A) and significantly lower mean expression values (in log_2_ TPM) (Figure 1B,C).

### 3.2. Selective Overexpression of TGFB2 mRNA in DIPG Tumor Specimens Is Associated with Augmented Expression of Transcription Factors Binding to Multiple TGFB2 Gene Promoter Sites

**Figure 2:**
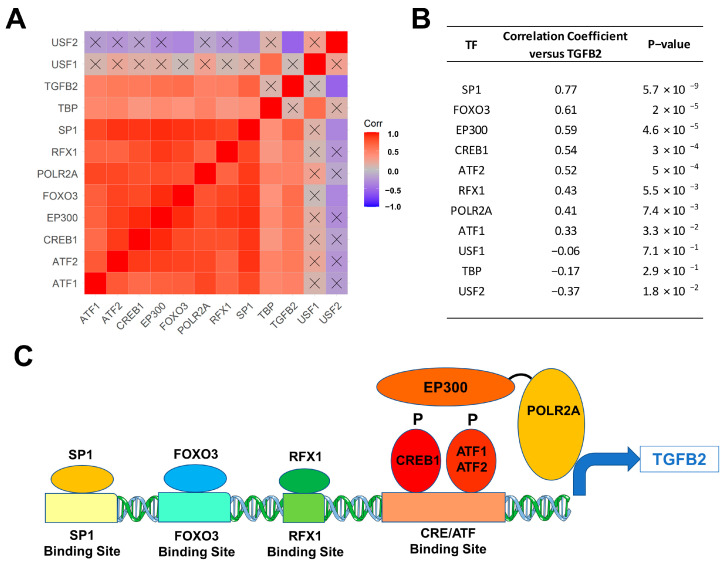
Correlation matrix of TGFB2 and transcription factors in 41 DIPG patients. Pairwise Pearson correlations were performed between the mRNA levels of TGFB2 and 11 transcription factors using log_2_-transformed TPM values obtained from cBioPortal for Cancer Genomics (https://pedcbioportal.kidsfirstdrc.org/). The correlation coefficients were calculated across 41 DIPG patients, depicted on the heatmap (**A**) ranging from positive correlations (red) to negative correlations (blue) and organized according to similarly expressed genes. The scale for the range of correlations is shown in the color bar, “Corr”. Of the 66 pairwise correlations, 48 were deemed statistically significant (*p* < 0.05 and FDR = 0.07; non-significant correlations are indicated with a black cross in the heat map). (**B**) The mRNA levels for 8 of 11 transcription factors (viz.: SP1, RFX1, POLR2A, FOXO3, EP300/transcription factor co-activator, CREB1, ATF2, and ATF1) exhibited statistically significant positive correlations with TGFB2 mRNA levels. SP1, FOXO3, and EP300 mRNA levels showed the most significant correlations with TGFB2 mRNA expression levels (*p* < 0.0001). (**C**) Depicted in the cartoon figure are the transcription factor proteins (oval shapes) bound to binding sites on the TGFB2 gene for these transcription factors (rectangles). Additionally, indicated are the recruitment of the transcription factor co-activator EP300 (E1A Binding Protein P300), that binds to phosphorylated forms of CREB1/ATF1/ATF2 and the bridging to the basal transcription machinery via the RNA Polymerase II Subunit A (POLR2A) to directly stimulate TGFB2 transcription.

We next sought to identify the molecular mechanism for the upregulation of TGFB2 mRNA expression levels in 41 DIPG tumor samples with a focus on transcription factors/DNA binding proteins that are known to augment TGFB2 gene expression. We examined the transcript-level expression of 11 transcription factors with enhancing activity on TGFB2 gene expression, namely ATF1, ATF2, CREB1, EP300, FOXO3, POLR2A, RFX1, SP1, TBP, USF1, and USF2 in relationship to TGFB2 mRNA levels [47,48,49,50,51,52]. The mRNA levels from 8 of these 11 transcription factors (viz.: SP1, RFX1, POLR2A, FOXO3, EP300, CREB1, ATF2, and ATF1) exhibited statistically significant positive correlations with TGFB2 mRNA levels (Figure 2). SP1, FOXO3, and EP300 showed the most significant correlations with TGFB2 mRNA expression levels (*p* < 0.0001).

**Figure 3:**
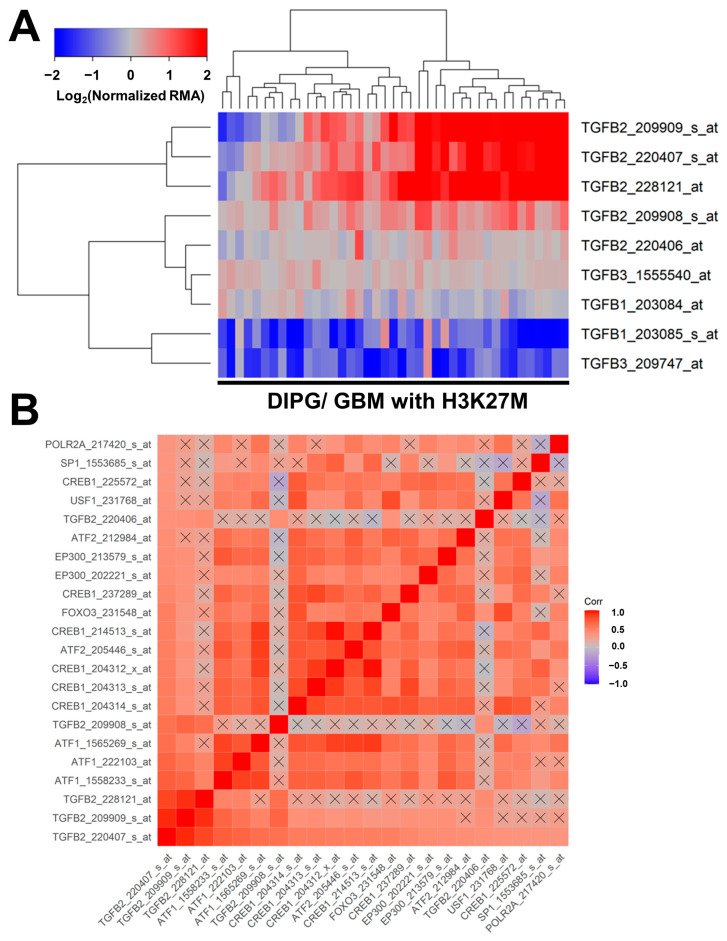
Correlated expression of TGFB2 mRNA and mRNA for specific transcription factors in an independent validation dataset. (**A**) Differential expression of mRNA levels for TGFB1, TGFB2, and TGFB3 are illustrated using a cluster representation of log_2_-transformed fold changes in 41 brain tumor specimens from pediatric patients with DIPG or H3K27M-mutant GBM. The expression levels were mean-centered to normal samples and depicted as log2-transformed normalized RMA values. The blue to red color in the heatmap indicates under-expression to over-expression, respectively, for the mRNA levels of TGFB1, TGFB2, and TGFB3. Co-regulated probesets are organized and depicted by dendrograms for both probesets (rows) and patients (columns). Significant increases in mRNA expressions were observed for three TGFB2 probesets: TGFB2_228121_at (2.7-fold increase, *p* = 0.006); TGFB2_209909_s_at (2.4-fold increase, *p* = 0.019); and TGFB2_220407_s_at (2.2-fold increase, *p* = 0.032). A significant decrease was observed in the mRNA expression of one of the TGFB1 probesets, TGFB1_203085_s_at (2.2-fold decrease, *p* = 0.028), and one of the TGFB3 probesets, TGFB3_209747_at (2.3-fold decrease, *p* = 0.026) (Table S1). (**B**) A total of 1640 correlations were performed for 41 probesets representing 11 transcription factors and TGFB2 mRNA. For 660 correlations, the *p*-values were less than 0.05 (FDR = 0.12), and 324 correlations exhibited *p*-values less than 0.001 (FDR = 0.005). The probeset TGFB2_220407_s_at exhibited the greatest number of positive correlations with the probesets for the 11 transcription factors and all four other TGFB2 probesets. There were 21 significant positive correlations with *p* < 0.05 (FDR = 0.047). Color-coded Pearson correlation coefficients for the most significant positive correlations with TGFB2_220407_s_at are depicted on the heatmap ranging from positive correlations (red) to negative correlations (blue) organized according to similarly expressed mRNA levels. The scale for the range of correlations is shown in the color bar, “Corr”. Non-significant correlations are indicated with a black cross in the heat map. The mRNA levels from 8 of the 11 transcription factors (viz.: SP1, USF1, POLR2A, FOXO3, EP300 (2 probesets), CREB1 (6 probesets), ATF2 (2 probesets), and ATF1 (3 probesets)) exhibited statistically significant positive correlations with TGFB2 mRNA levels (Table S2).

Similar results were obtained in an independent validation dataset of microarray-based mRNA levels for TGFB1, TGFB2, and TGFB3, and their correlated expression with specific transcription factors in tumor specimens from 41 pediatric patients with DIPG (*n* = 29) or H3K27M-mutant GBM (Figure 3). Significant increases in mRNA expression levels were observed for three TGFB2 probesets: TGFB2_228121_at (2.7-fold increase, *p* = 0.006); TGFB2_209909_s_at (2.4-fold increase, *p* = 0.019); and TGFB2_220407_s_at (2.2-fold increase, *p* = 0.032) (Figure 3, Panel A; Table S1). A significant decrease was observed in the mRNA expression of one of the TGFB1 probesets, TGFB1_203085_s_at (2.2-fold decrease, *p* = 0.028), and one of the TGFB3 probesets, TGFB3_209747_at (2.3-fold decrease, *p* = 0.026) (Table S1). The probeset TGFB2_220407_s_at exhibited the greatest number of positive correlations with the probesets for the 11 transcription factors and all four other TGFB2 probesets (21 significant positive correlations with *p* < 0.05 (FDR = 0.047) (Figure 3, Panel B). The mRNA levels from 8 of the 11 transcription factors [viz.: SP1, USF1, POLR2A, FOXO3, EP300 (2 probesets), CREB1 (6 probesets), ATF2 (2 probesets), and ATF1 (3 probesets)] exhibited statistically significant positive correlations with TGFB2 mRNA levels (Figure 3, Panel B; Table S2).

### 3.3. Amplified Expression of TGFB2 mRNA but Not TGFB1 or TGFB3 mRNA, Is Associated with Shorter OS and PFS in DIPG Patients

**Figure 4:**
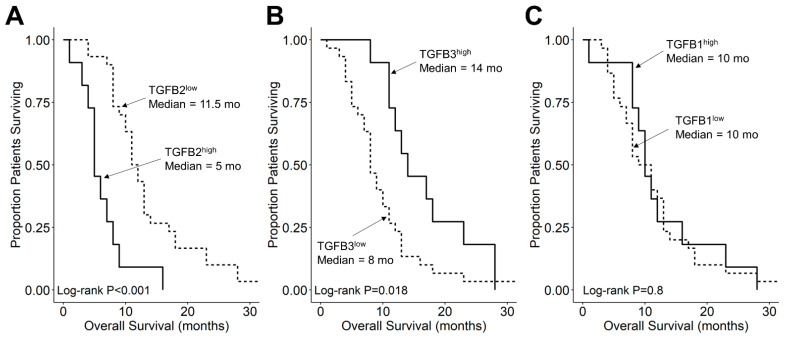
Amplified expression of TGFB2 is associated with shorter OS in DIPG patients. Archived survival outcome data for 41 DMG patients, including 11 DIPG patients with unknown H3K27M mutational status, 4 H3K27M-mutant DIPG patients and 26 H3K27M-mutant DMG patients, whose brain tumors were localized to pons/brainstem, obtained from cBioPortal for Cancer Genomics (https://pedcbioportal.kidsfirstdrc.org/) was combined with RNAseq-based mRNA expression data for of TGFB2 (**A**), TGFB3 (**B**), and TGFB1 (**C**) to assess the potential impact of the respective TGFB isoform levels on OS. Log_2_-transformed, TPM-normalized RNAseq values were rank-ordered according to expression level for each TGFB isoform. Patients whose expression levels for a given TGFB isoform was greater than or equal to the upper quartile (solid line) were compared to the remaining patients (dashed line). See the legend of Figure 1 for the TGFB isoform levels in the analyzed patient subsets. (**A**) The mean expression level for TGFB2 in the 11-patient TGFB2^high^ subset was 6.19 ± 0.24 (median, range = 5.81, 5.23–7.63). By comparison, the mean TGFB2 expression level for the 30-patient TGFB2^low^ subset was 3.22 ± 0.23 (median, range = 3.48, −0.42–5.07). Patients with high TGFB2 mRNA expression (TGFB2^high^) exhibited a significantly worse OS outcome than rest of the patients (TGFB2^low^) (median OS for TGFB2^high^ subset: 5 months, 95% CI: 5–NA months, 11 events, N = 11); median OS for TGFB2^low^ subset: 11.5 months, 95% CI: 10–14 months, 30 events, N = 30; log-rank chi-square = 16.2, *p* = 5.6 × 10^−5^). (**B**) The mean expression level for TGFB3 in the 11-patient TGFB3^high^ subset was 4.7 ± 0.2 (median, range = 4.6, 4–5.6). By comparison, the mean TGFB3 expression level for the 30-patient TGFB3^low^ subset was 3 ± 0.1 (median, range = 3.1, 1.5–4). Patients with high TGFB3 expression (TGFB3^high^) (median OS: 14 months, 95% CI: 12–NA months, 11 events, N = 11) exhibited a significantly better OS outcome than TGFB3^low^ patients (median OS: 8 months, 95% CI: 7–11 months, 30 events, N = 30; log-rank chi-square = 5.6, *p* = 0.018). (**C**) The mean expression level for TGFB1 mRNA in the 11-patient TGFB1^high^ subset was 4.8 ± 0.1 (median, range = 4.8, 4.5–5.4). By comparison, the mean TGFB1 expression level for the 30-patient TGFB1^low^ subset was 3.8 ± 0.1 (median, range = 3.9, 1–4.5). Patients with high TGFB1 expression (TGFB1^high^) (median OS = 10 months, 95% CI: 9–NA months, 11 events, N = 11) exhibited similar OS outcomes when compared to the rest of the patients (TGFB1^low^) (median OS = 10 months, 95% CI: 8–13 months, 30 events, N = 30; log-rank chi-square = 0.1, *p* = 0.8).

**Figure 5:**
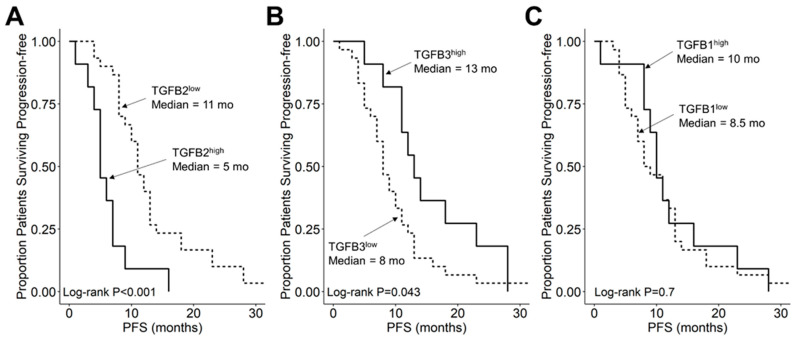
Amplified expression of TGFB2 is associated with shorter PFS in DIPG and DMG patients. Archived clinical outcome data for 41 DMG patients, including 11 DIPG patients with an unknown H3K27M mutational status, 4 H3K27M-mutant DIPG patients and 26 H3K27M-mutant DMG patients, whose brain tumors were localized to brainstem/pons, obtained from cBioPortal for Cancer Genomics (https://pedcbioportal.kidsfirstdrc.org/) was combined with RNAseq-based mRNA expression data for TGFB2 (**A**), TGFB3 (**B**), and TGFB1 (**C**) to assess the potential impact of the respective TGFB isoform levels on PFS. For patients with missing information regarding progression of disease, death was used as the first event when evaluating the PFS outcome. Log_2_-transformed, TPM-normalized RNAseq values were rank-ordered according to expression level for each TGFB isoform. Patients whose expression levels for a given TGFB isoform was greater than or equal to the upper quartile (solid line) were compared to the remaining patients (dashed line). (**A**) TGFB2^high^ patients with exhibited a significantly worse PFS outcome than rest of the patients (median PFS for TGFB2^high^ subset: 5 (95% CI: 5–NA) months, 11 events, N = 11); median PFS for TGFB2^low^ subset = 11 months, 95% CI: 10–13 months, 30 events, N = 30; log-rank chi-square = 14.7, *p* = 1.3 × 10^−4^). (**B**) TGFB3^high^ patients (median PFS = 13 months, 95% CI: 11–NA months, 11 events, N = 11) exhibited a significantly better PFS outcome than TGFB3^low^ patients (median PFS = 8 months, 95% CI: 7–11 months, 30 events, N = 30; log-rank chi-square = 4.1, *p* = 0.043). (**C**) TGFB1^high^ patients (median PFS = 10 months, 95% CI: 9–NA months, 11 events, N = 11) and TGFB1^low^ patients exhibited similar OS outcomes (median PFS = 8.5 months, 95% CI: 7–13 months, 30 events, N = 30; log-rank chi-square = 0.2, *p* = 0.7).

We compared the survival outcomes of DIPG patients with TGFB2 mRNA expression levels greater than or equal to the upper quartile (TGFB2^high^) to the treatment outcomes of the remaining DIPG patients (TGFB2^low^). The mean expression level for TGFB2 in the 11-patient TGFB2^high^ subset was 6.2 ± 0.2 (median, range = 5.8, 5.2–7.6). By comparison, the mean TGFB2 mRNA expression level for the 30-patient TGFB2^low^ subset was 3.2 ± 0.2 (median, range = 3.5, −0.4–5.1). TGFB2^high^ DIPG patients exhibited a significantly worse OS outcome than TGFB2^low^ DIPG patients (median OS for TGFB2^high^ subset: 5 months, 95% CI: 5–NA months, 11 events, N = 11); median OS for TGFB2^low^ subset: 11.5 months, 95% CI: 10–14 months, 30 events, N = 30; log-rank chi-square = 16.2, *p* = 5.6 × 10^−5^) (Figure 4). The PFS outcome was also significantly worse for the TGFB2^high^ subset (median PFS for TGFB2^high^ subset: 5 (95% CI: 5–NA) months, 11 events, N = 11) vs. median PFS for TGFB2^low^ subset = 11 months, 95% CI: 10–13 months, 30 events, N = 30 (log-rank chi-square = 14.7, *p* = 1.3 × 10^−4^) (Figure 5 and Figure S1). In contrast to the poor prognostic impact of high TGFB2 mRNA expression, high TGFB3 mRNA expression was associated with significantly better OS and PFS outcomes, and high TGFB1 mRNA expression had no statistically significant effect on OS or PFS outcomes (Figure 4 and Figure 5).

We also separately evaluated the OS outcome data for the 30 H3K27M-mutant DIPG patients excluding the 11 DIPG patients with an unknown H3K27M mutational status. TGFB2^high^ patients within the 30-patient subset had a shorter time to death than the remaining patients, similar to the TGFB2^high^ patients in the 41-patient full analysis set, which included 11 DIPG patients with an unknown H3K27M mutational status (Figure S2). However, the difference did not reach statistical significance in the smaller subset, which is likely due to the reduction of power to detect a statistical difference arising from the broader distribution of OS outcomes and smaller sample size (Figure S2). Notably, as with the full analysis set of 41 patients (Figure S3, Panel A), TGFB2^high^ patients in the 30-patient subset were characterized by early failures and a significantly worse survival outcomes within 10 months (Figure S3, Panel B). The median survival for TGFB2^high^ patients within the 30-patient H3K27M-mutant subset was 6.5 months (95% CI = 5-NA months, 6 events, N = 8) which was significantly shorter than the median survival for the remaining patients (median >10 months, 7 events, N = 22; log-rank chi-square = 5.5, *p*-value = 0.019) (Figure S3, Panel B).

### 3.4. TGFB2 Expression Level Does Not Affect OS or PFS in Pediatric DMG Patients Whose Tumor Is Not Located in Pons/Brainstem

We compared the survival outcomes of non-DIPG DMG patients whose tumor location was outside the pons/brainstem with TGFB2 mRNA expression levels greater than or equal to the upper quartile (TGFB2^high^) to the treatment outcomes of the remaining non-DIPG DMG patients (TGFB2^low^). TGFB2^high^ patients (median OS = 12.5 months, 95% CI: 9–NA months, 10 events) exhibited a similar OS outcome when compared to TGFB2^low^ patients (median OS = 11 months, 95% CI: 2–NA months, 8 events; log-rank chi-square = 0.2, *p* = 0.6) (Figure S4). TGFB2^high^ and TGFB2^low^ non-DIPG DMG patients also had very similar PFS outcomes (Figure S5). Similarly, no statistically significant differences in OS or PFS outcomes were found in TGFB1^high^ vs. TGFB1^low^ or TGFB3^high^ vs. TGFB3^low^ non-DIPG DMG subset comparisons (Figures S4 and S5). Since the activation of TGFB signaling pathways requires binding of TGFB to the TGF-β receptor II (TGF-βRII), which recruits TGF-βRI into a heterotetrameric complex [31], we next sought to determine if the observed lack of a prognostic effect of TGFB2^high^ status could be due to decreased TGFB2 receptor expression levels. None of the TGFB2 receptors exhibited a lower expression level in tumor samples from non-DIPG DMG patients (N = 19) compared to the tumor samples from DIPG patients (N = 41) to explain the observed lack of prognostic significance of higher TGFB2 mRNA levels in this patient group (Figure S6). We noticed that the DIPG patients were on average younger (mean age at diagnosis in months = 7.0 ± 0.4; median = 6; range = 2–14) than the non-DIPG DMG patients (mean age at diagnosis in months = 10.5 ± 0.7; median = 11; range = 5–17) (*p* = 0.0003).

### 3.5. TGFB2 mRNA Expression Level Does Not Affect OS or PFS in Pediatric GBM Patients

**Figure 6:**
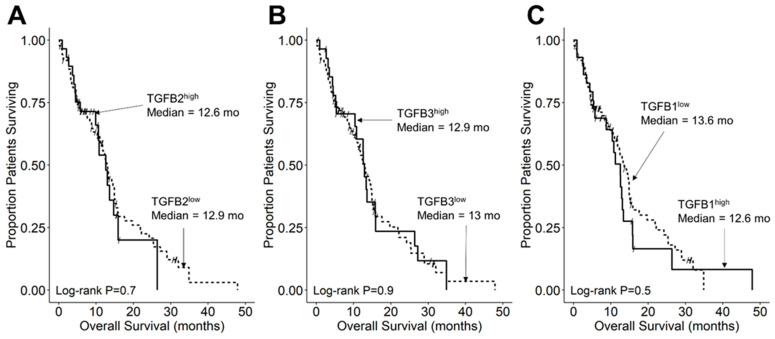
TGFB2 mRNA expression level does not affect OS in pediatric GBM patients. Archived OS data for 116 GBM patients obtained from cBioPortal for Cancer Genomics (https://pedcbioportal.kidsfirstdrc.org/) was combined with RNAseq-based mRNA expression data for TGFB2 (**A**), TGFB3 (**B**), and TGFB1 (**C**) to assess the potential impact of the respective TGFB isoform levels on OS. Log_2_-transformed, TPM-normalized RNAseq values were rank-ordered according to expression level for each TGFB isoform. Patients whose expression levels for a given TGFB isoform was greater than or equal to the upper quartile (solid line) were compared to the remaining patients (dashed line). See the legend of Figure 3 for the TGFB isoform levels in the analyzed patient subsets. (**A**) The mean expression level for TGFB2 in the 29-patient TGFB2^high^ subset was 12.67 ± 0.1 (median, range = 12.63, 11.97–13.7). By comparison, the mean expression level for TGFB2 in the 87-patient TBFB2^low^ subset was 10.37 ± 0.1 (median, range = 10.49, 8.17–11.94). Patients in the TGFB2^high^ (N = 29) and TGFB2^low^ (N = 87) subsets exhibited very similar OS outcomes (median for TGFB2^high^: 12.6 (95% CI: 9.9–NA) months, 17 events; median for TGFB2^low^: 12.9 (95% CI: 11.24–14.95) months, 65 events; log-rank chi-square = 0.2, *p* = 0.7). (**B**) The mean expression level for TGFB3 in the 29-patient TGFB3^high^ subset was 11.39 ± 0.1 (median, range = 11.25, 10.63–12.88). By comparison, the mean expression level for TGFB3 in the 87-patient TBFB3low subset was 9.4 ± 0.08 (median, range = 9.56, 7.14–10.58). Patients in the TGFB3^high^ and TGFB3^low^ subsets exhibited very similar OS outcomes (median for TGFB3^high^: 12.9 (95% CI: 10.41–26.38) months, 20 events); median for TGFB3^low^: 13 (95% CI: 11–14.95) months, 62 events; log-rank chi-square = 0, *p* = 0.9). (**C**) The mean expression level for TGFB1 in the 29-patient TGFB1^high^ subset was 12.08 ± 0.08 (median, range) = 11.92, 11.58–13.13). By comparison, the mean expression level for TGFB1 in the 87-patient TBFB1^low^ subset was 10.53 ± 0.09 (median, range = 10.71, 7.5–11.56). TGFB1^high^ patients (median: 12.6 (95% CI: 8.8–15.8) months, 22 events; N = 29) and TGFB1^low^ patients (median: 13.6 (95% CI: 11.7–15.4) months, 60 events) exhibited similar OS outcomes (log-rank chi-square = 0.5, *p* = 0.5).

We compared the survival outcomes of pediatric GBM patients whose TGFB2 mRNA expression levels were greater than or equal to the upper quartile (TGFB2^high^) to the treatment outcomes of the remaining pediatric GBM patients (TGFB2^low^). The mean expression level for TGFB2 in the 29-patient TGFB2^high^ pediatric GBM subset was 12.7 ± 0.1 (median, range = 12.6, 12–13.7). By comparison, the mean expression level for TGFB2 in the 87-patient TBFB2^low^ pediatric GBM subset was 10.4 ± 0.1 (median, range = 10.5, 8.2–12). Patients in the TGFB2^high^ (N = 29) and TGFB2^low^ (N = 87) subsets exhibited very similar OS outcomes (median for TGFB2^high^: 12.6 (95% CI: 9.9–NA) months, 17 events; median for TGFB2^low^: 12.9 (95% CI: 11.2–15) months, 65 events; log-rank chi-square = 0.2, *p* = 0.7) (Figure 6). TGFB2^high^ and TGFB2^low^ pediatric GBM patients also had very similar PFS outcomes (Figure S7). Similarly, no differences in OS or PFS outcomes were found in TGFB1^high^ vs. TGFB1^low^ or TGFB3^high^ vs. TGFB3^low^ pediatric GBM subset comparisons (Figure 5 and Figure S7). None of the TGFB2 receptors exhibited a lower expression level in tumor samples from GBM patients vs. DIPG/DMP patients to explain the observed lack of prognostic significance of higher TGFB2 mRNA levels in this patient population. To the contrary, GBM patients exhibited significantly higher levels of all three receptors compared to DIPG patients (Figure S8). The TGFB2 mRNA levels in GBM patients were higher than those of DIPG patients; the mean expression level for TGFB2 mRNA even in the 11-patient TGFB2^high^ DIPG subset was 6.19 ± 0.24 (median, range = 5.81, 5.23–7.63), which is lower than the TGFB2 mRNA levels of TGFB2^low^ GBM patients (10.4 ± 0.1; median, range = 10.5, 8.2–12). The absence of a truly TGFB2^low^ subset in GBM patients might have precluded an accurate evaluation of the prognostic effect of high TGFB2 mRNA levels. We also noticed that the DIPG patients were on average younger (mean age at diagnosis in months = 7.0 ± 0.4; median = 6; range = 2–14) than the GBM patients (mean age at diagnosis in months = 60.0 ± 1.2; median = 60.4; range = 21–89.3) (*p* < 0.0001).

## 4. Discussion

In the present study, we examined the prognostic significance of TGFB2^high^ status in newly diagnosed pediatric DIPG patients. Notably, TGFB2^high^ (but not TGFB1^high^ or TGFB3^high^) patients had significantly worse survival outcomes with substantially shorter OS times. RNAseq-based high TGFB2 mRNA level was an indicator of poor prognosis for DIPG patients, but not for pediatric GBM patients or pediatric DMG patients with tumor locations outside of the pons/brainstem. The lack of an adverse prognostic effect for higher TGFB2 mRNA expression levels in the latter patient populations was not due to lower expression levels of TGFB2 receptors. It is noteworthy that the TGFB2 mRNA levels in pediatric GBM patients were markedly higher than those of DIPG patients. The absence of a truly TGFB2^low^ subset in GBM patients might have precluded an accurate evaluation of the favorable prognostic effect of low TGFB2 mRNA levels.

Our study fills a significant gap in our understanding of the clinical significance of high TGFB2 expression in pediatric high-grade gliomas. We propose that the TGFB2-promoted invasive growth of DIPG cells, their TGFB2-associated radiation resistance, and possibly TGFB2-mediated restrictions of the cellular anti-glioma immunity within the TME contribute to the observed adverse impact of high TGFB2 levels on the survival outcomes of DIPG patients.

In a recent study that employed a microarray-based gene expression platform, we found that TGFB2 transcript, but not TGFB1 or TGFB3 levels were selectively amplified in primary tumor samples from 29 pediatric DIPG patients compared to normal samples and primary tumor samples from grade low-grade glioma patients [39]. In the present study, we compared the RNAseq-based TGFB2 mRNA levels in primary DIPG tumor samples and normal control pons samples. Notably, tumor samples from DIPG patients expressed significantly higher levels of TGFB2 mRNA than control pons samples, but the TGFB1, as well as TGFB3 mRNA levels in DIPG samples were significantly lower than in the control pons samples. These results generated using the RNAseq-based mRNA data confirm and significantly expand our previous results obtained using the microarray platform. We hypothesized that the augmented expression of the TGFB2 gene in DIPG samples might be due to the enhanced expression of transcription factors that upregulate TGFB2 mRNA expression [47,48,49,50,51,52,53]. This hypothesis was supported by our finding of a strong positive correlation (*p* < 0.0001) between the mRNA levels of several such transcription factors, including SP1, FOXO3, and EP300 and TGFB2 mRNA levels in DIPG samples. We obtained similar results in an independent validation dataset of microarray-based mRNA levels for TGFB1, TGFB2, and TGFB3, and their correlated expression with specific transcription factors in tumor specimens from 41 pediatric patients with DIPG (N = 29) or H3K27M-mutant GBM.

**Figure 7:**
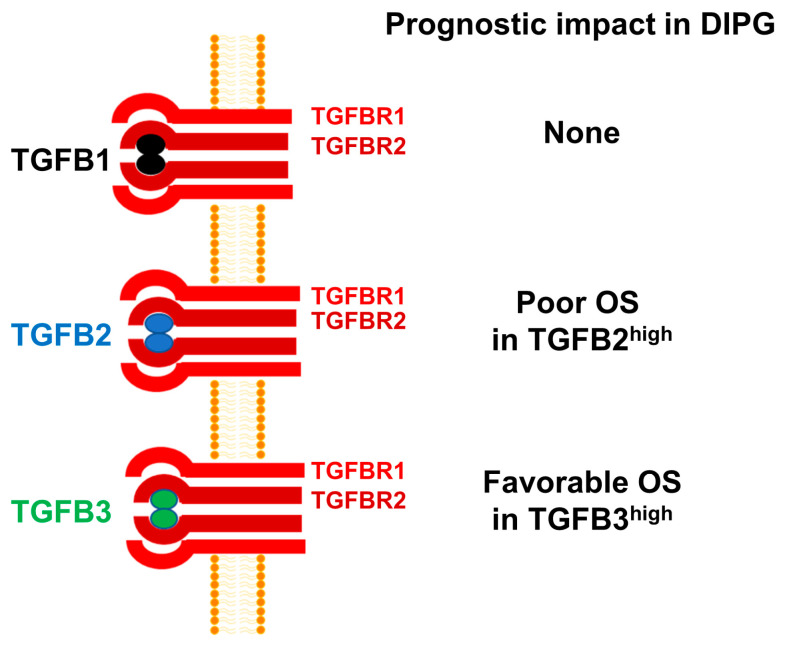
TGFB2 mRNA expression is selectively amplified and associated with poor OS in pediatric DIPG. mRNA levels for TGFB2 were selectively amplified in primary brain tumor specimens from DIPG patients, whereas the mRNA levels for TGFB1 and TGFB3 were not. High levels of TGFB2 mRNA expression was associated with poor OS. By contrast, high TGFB1 mRNA expression had no prognostic value and high TGFB3 mRNA expression was associated with favorable OS.

There are three isoforms of TGFB within the TGFB superfamily of genes, namely TGFB1, TGFB2, and TGFB3 [30,54]. Despite a >70% sequence homology, the structure and biological functions of the TGFB isoform TGFB3 differs from those of TGFB1 and TGFB2 [55]. Notably, TGFB3-knockout mice are uniquely different from TGFB1-knockout or TGFB2-knockout mice [56,57,58]. Furthermore, TGFB3 has also been reported to exert a cancer-preventive effect in non-clinical models of tumor development as well as human subjects [55]. Additionally, high TGFB3 expression has been reported to have a favorable prognostic effect in breast cancer, ovarian cancer, and colon cancer. However, TGFB3 expression has been correlated with a poor prognosis in osteosarcoma [59]. TGFB3 expression has also been detected in human glioma cells and depletion of TGFB3 mRNA using TGFB3-specific antisense oligonucleotides decreased the invasiveness of human glioma cells in vitro, but its role in pathobiology or prognosis of high-grade gliomas remains unknown [60]. In the present study, high TGFB3 expression was identified as a favorable prognostic indicator which contrasts with its reported adverse prognostic role in breast cancer. Our observation expands our current knowledge and provides new insights regarding the multifunctional role of TGFB3 [61]. Based on the results presented herein, we propose a model according to which amplified TGFB2 mRNA expression is associated with poor prognosis and OS in DIPG (Figure 7).

Inhibition of the TGFB signaling pathway is being explored as a clinical strategy for the treatment of patients with advanced or poor prognostic subtypes of cancer [62,63,64]. Several modalities have been developed, including small-molecule kinase inhibitors, monoclonal antibodies, and RNA interference (RNAi) therapeutics [62,63,64]. RNAi therapeutics targeting TGFB2, including S-ODN, may disrupt the TGFB2-linked signaling networks that promote the proliferation and survival of TGFB2^high^ DIPG cells, lift TGFB2-induced suppression of anti-DIPG immunity, as well as block the Treg-mediated tumor tolerance [40]. We previously reported that OT-101, a TGFB2-targeting S-ODN, exhibited promising single-agent clinical activity in adult patients with recurrent or refractory glioblastoma and anaplastic astrocytoma when administered intratumorally via CED [37]. Of the 77 high-grade glioma patients in the efficacy population, 26 had a favorable response, including 19 patients who had a CR or PR and 7 patients who had a stable disease with a longer than 6-month duration [37]. The median PFS for this subset was 1109 days and their OS was 1280 days [37]. The observed poor prognosis of newly diagnosed TGFB2^high^ DIPG patients, as reported here, supports the notion that further exploration of the clinical potential of TGFB2-targeting RNAi therapeutics in TGFB2^high^ DIPG patients is warranted. It is noteworthy that CED catheters have been used in DIPG patients for intratumoral delivery of therapeutic agents in an attempt to bypass the blood–brain barrier and achieve higher intratumor concentrations while mitigating the risk of systemic toxicity [65,66,67]. However, the apparent requirement for months-long intratumoral administration of the anti-TGFB2 S-ODN OT-101 for achieving objective responses in high-grade adult glioma patients [37], combined with the practical challenges of prolonged use of implanted CED catheters or repeated replacement surgeries for CED catheters at this difficult anatomic location, indicates that discovery of innovative delivery methods and/or formulation strategies will likely be required for TGFB2-targeting RNAi therapeutics to be effective in DIPG patients. Other BBB-disrupting or BBB-bypassing strategies might also be potentially helpful in effective tumor delivery of TGFB2-directed RNAi therapeutics in DIPG patients [68,69,70,71].

The present study has significant limitations including the missing H3K27M mutational status for 11 of the 41 DIPG patients, and the bioinformatics-based analyses used without additional supportive laboratory testing of TGFB2 mRNA levels and mRNA levels of correlated transcription factors using other assay platforms such as quantitative RT-PCR and validated immunohistochemistry tests that are not available. Another limitation of the study relates to the absence of archived patient-specific treatment information in the database which prevented a detailed analysis of the cause of poor OS outcomes in relationship to the treatments used and formal exclusion of a patient selection bias that resulted from differences in treatments. This is especially important for our unexpected finding that TGFB2^high^ status had no apparent prognostic impact in non-DIPG DMG patients whose tumor was not located in the pons/brainstem. While the DIPG patients were younger than the non-DIPG DMG patients, no age-related changes in the TGFB2 responsiveness of high-grade pediatric glioma cells or tumor microenvironment composition in DMG have been reported to suggest the observations were in part age-related. In this context, it is noteworthy that DIPG patients were also younger than GBM patients. Because freshly produced TGFB2 is in an inactive form bound to the latency-associated peptide (LAP) when first released into the TME, a further limitation of the current study is the lack of biochemical data to show that increased mRNA levels for TGFB2 are associated with increased levels of active TGFB2 protein and TGFB2 signaling pathway activity [72]. A hypothesis-testing prospective validation study will be necessary to confirm the poor prognostic effect of the TGFB2^high^ status, preferably with supporting RT-qPCR and RNAseq data in a larger DIPG patient population treated according to a contemporary standard of care regimen.

## 5. Conclusions

We present new evidence that amplified expression of TGFB2 mRNA in pediatric DIPG and H3K27M-mutant GBM is correlated with upregulated mRNA expression for several transcription factors/DNA binding proteins that are known to augment TGFB2 gene expression. Our findings provide the first evidence that high level TGFB2 mRNA expression is associated with a poor treatment outcome in DIPG. The reported results also support the notion that further evaluation of the clinical potential of new strategies targeting TGFB2 mRNA in pediatric DIPG is warranted.

## Data Availability

We used publicly available archived gene expression profiling datasets to evaluate the expression levels of TGF isoforms in tumor samples from pediatric high-grade glioma patients. We downloaded clinical metadata and RNAseq-based mRNA expression data for 41 pediatric DMG patients and 116 pediatric GBM patients regarding TGFB isoforms TGFB1, TGFB2, and TGFB3 from the genomic data set repository stored in the cBioPortal for Cancer Genomics (https://pedcbioportal.kidsfirstdrc.org/ (accessed on 4 November 2022)) using the interactive Web interface with full filtering functionality provided by the portal. RNAseq-based mRNA expression values for normal pons specimens (rna_tissue_hpa.tsv.zip) were downloaded from https://www.proteinatlas.org/about/download (accessed on 19 December 2022). The original contributions presented in the study are included in the article/Supplementary Material. Further inquiries can be directed to the corresponding author.

## Supplementary Materials

The following supporting information can be downloaded at: https://www.mdpi.com/article/10.3390/cancers15061676/s1, Figure S1. PFS and OS outcomes of 41 DIPG patients in relationship to TGFB2 mRNA expression levels, Figure S2. Amplified expression of TGFB2 is associated with shorter OS in DIPG patients, Figure S3. TGFB2^high^ status in DIPG is associated with early treatment failures and death, Figure S4. TGFB2 expression level does not affect OS in DMG patients with tumor location outside pons/brainstem, Figure S5. TGFB2 expression level does not affect PFS in DMG patients with tumor location outside pons/brainstem, Figure S6. Expression of receptors for TGFB2 (TGFBR1/2/3) in Pons and Non-Pons DMG patients, Figure S7. TGFB2 expression level does not affect PFS in pediatric GBM patients, Figure S8. Expression of receptors for TGFB2 (TGFBR1/2/3) in DIPG and GBM patients, Table S1. Increased TGFB2 mRNA expression levels in pediatric patients with DIPG or H3K27M-mutant GBM, Table S2. TGFB2_220407_s_at mRNA probeset correlations with transcription factors in pediatric patients with DIPG or H3K27M-mutant GBM.
