# Supplementary material for: High Intra-Tumor Transforming Growth Factor Beta 2 Level as a Predictor of Poor Treatment Outcomes in Pediatric Diffuse Intrinsic Pontine Glioma
Source: Cancers (Basel). 2023 Mar 9;15(6):1676. doi: 10.3390/cancers15061676 (PMC10046593; doi:10.3390/cancers15061676)
Supplement: Supplementary file 1 [file cancers-15-01676-s001.zip › cancers-2174803-supplementary.pdf]

Supplemental Figures and Tables

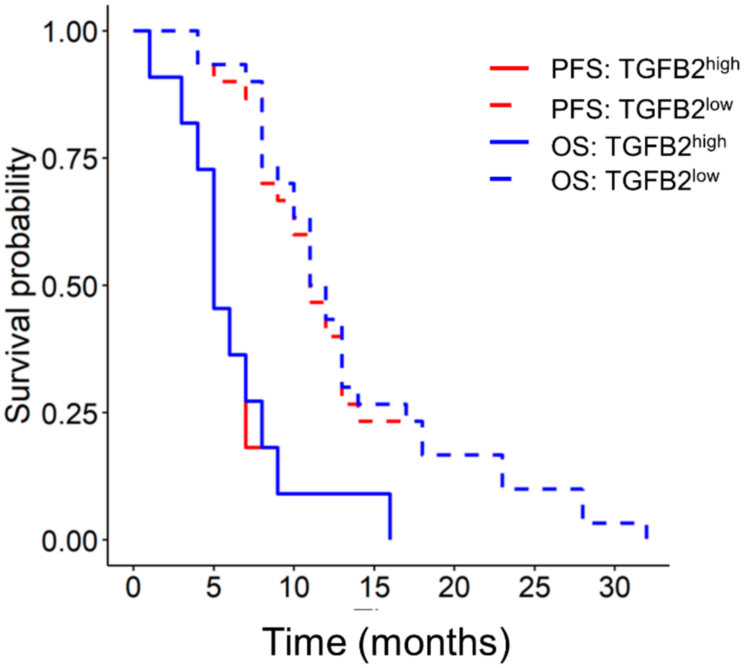

Figure S1. PFS and OS outcomes of 41 DIPG patients in relationship to TGFB2 mRNA expression levels. Depicted is an overlay of OS and PFS curves from Figure 4, Panel A and Figure 5, Panel A.

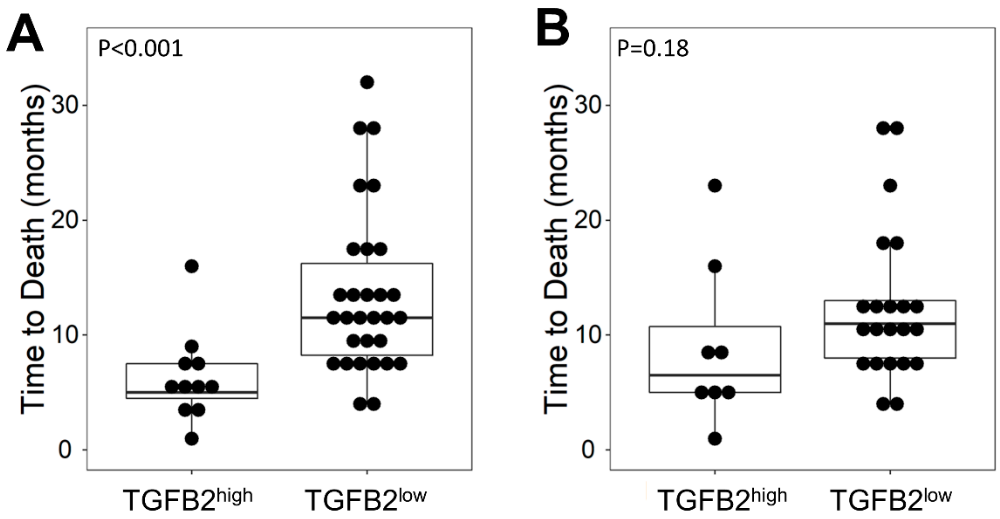

Figure S2. Amplified expression of TGFB2 is associated with shorter OS in DIPG patients. We compared the OS outcome data for the 30 H3K27M-mutant DIPG patients with the OS outcome data for the full analysis set of 41 patients, which included 11 DIPG patients with an unknown H3K27M mutational status. [A] Depicted are box plots of time to death (in months) for the full analysis set of 41 patients. $TGFB2^{high}$ patients exhibited a significantly worse OS outcome than the remaining patients ($TGFB2^{low}$). The median OS for $TGFB2^{high}$ patients was 5 months (95% CI: 5 - NA months, 11 events, N=11). By comparison, the median OS for $TGFB2^{low}$ was 11.5 months (95% CI: 10 - 14 months, 30 events, N=30). This difference was statistically significant (log-rank chi-square = 16.2, P = 5.6 x 10^{-5}). [B] Depicted are box plots of time to death (in months) for the 30 H3K27M-mutant DIPG patients. $TGFB2^{high}$ patients exhibited a worse OS outcome compared to $TGFB2^{low}$ patients. The median OS for $TGFB2^{high}$ patients was 6.5 months (95% CI = 5 - NA months; 8 events, N = 8). By comparison, the median OS for $TGFB2^{low}$ patients was 11 months (95% CI = 10 - 13 months; 22 events, N = 22). This difference was not statistically significant (log-rank chi-square = 1.78, P-value = 0.18).

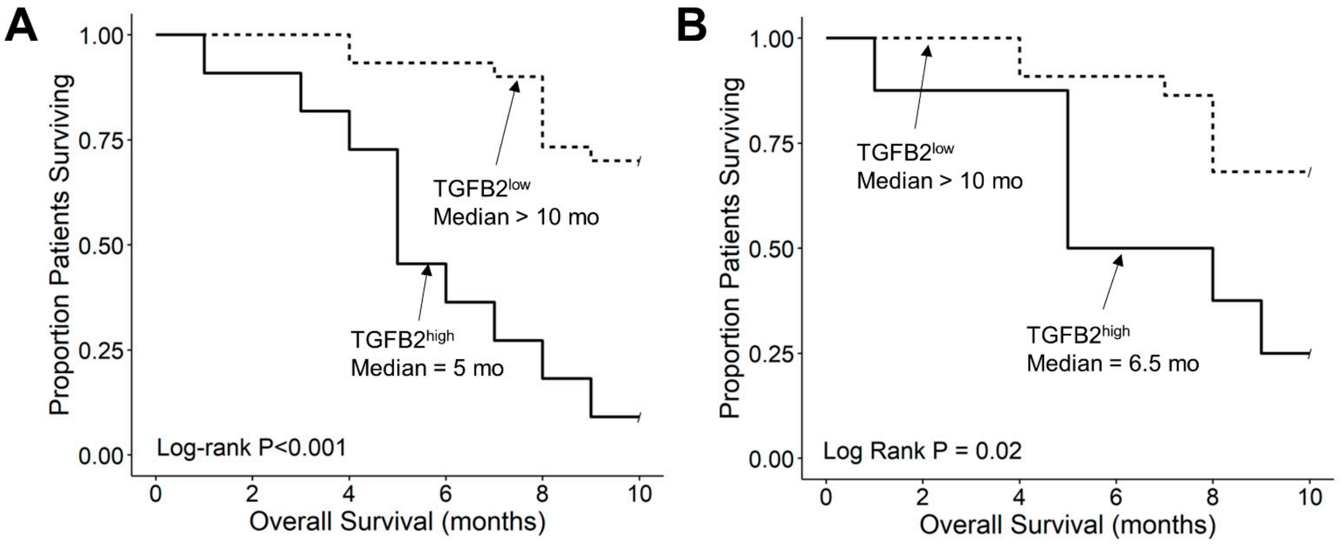

Figure S3. TGFB2^{high} status in DIPG is associated with early treatment failures and death. The OS outcomes of TGFB2^{high} and TGFB2^{low} patients were compared for early time points up to 10 months by censoring all events occurring at ≥10 months. [A] OS curves for the full analysis set of 41 DIPG patients, which included 11 DIPG patients with unknown H3K27M mutational status. TGFB2^{low} patients (median > 10 months; N = 30; 9 events) exhibited a statistically significant longer OS outcome than TGFB2^{high} patients (median = 5; 95% CI = 5 - NA months; N = 11; 10 events ; log-rank chi-square = 20.9, P-value < 0.001). [B] OS curves for 30 H3K27M-mutant DIPG patients. TGFB2^{low} patients (median > 10 months; N = 22; 7 events) exhibited a statistically significantly longer OS outcome than TGFB2^{high} patients (median = 6.5; 95% CI = 5 - NA months; N = 8; 6 events; log-rank chi-square = 5.5, P-value = 0.019).

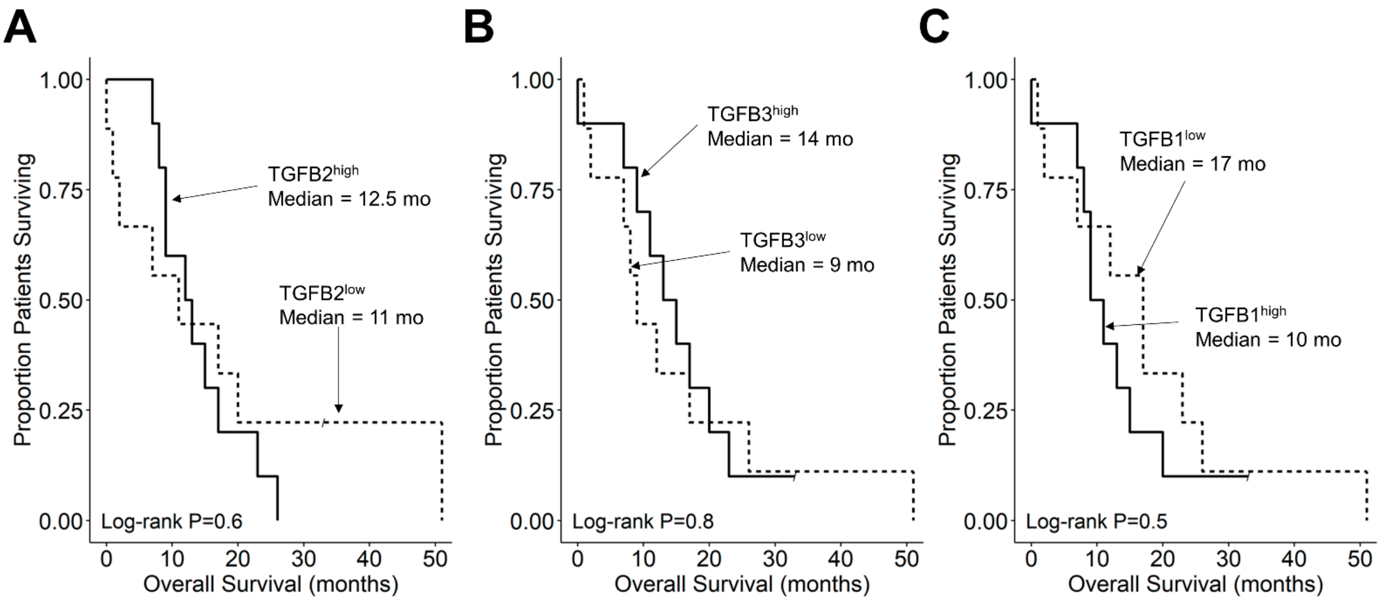

Figure S4. TGFB2 expression level does not affect OS in DMG patients with tumor location outside pons/brainstem. Archived OS data for 19 H3K27M-mutant DMG patients, whose brain tumors were not localized to the brainstem/pons, obtained from cBioPortal for Cancer Genomics (https://pedcbioportal.kidsfirstdrc.org/) was combined with RNAseq-based mRNA expression data for TGFB2 **[A]**, TGFB3 **[B]**, and TGFB1 **[C]** to assess the potential impact of the respective TGFB isoform levels on OS. Log_2-transformed, TPM-normalized RNAseq values were rank-ordered according to expression level for each TGFB isoform. Patients whose expression levels for a given TGFB isoform was greater than or equal to the median (solid line) were compared to the remaining patients (dashed line). **[A]** The mean expression level for TGFB2 in the 10-patient $TGFB2^{high}$ subset was 5.4 ± 0.18 (median, range = 5.13, 4.86 - 6.32, N=10). By comparison, the mean TGFB2 expression level for the 9-patient $TGFB2^{low}$ subset was 3.95 ± 0.31 (median, range = 4.32, 2.06 - 4.83). $TGFB2^{high}$ patients (median OS = 12.5 months, 95% CI: 9 - NA months, 10 events) exhibited a similar OS outcome when compared to $TGFB2^{low}$ patients (median OS = 11 months, 95% CI: 2 - NA months, 8 events; log-rank chi-square = 0.2, P = 0.6). [**B**] The mean expression level for TGFB3 in the 10-patient $TGFB3^{high}$ subset was 4.38 ± 0.24 (median , range = 4.17, 3.34 - 6.07). By comparison, the mean TGFB3 expression level for the 9-patient $TGFB3^{low}$ subset was 2.32 ± 0.25 (median, range = 2.17, 0.82 - 3.08). $TGFB3^{high}$ patients (median OS = 14 months, 95% CI: 9 - NA months, 9 events) exhibited a statistically insignificant better OS outcome when compared to $TGFB3^{low}$ patients (median OS = 9 months, 95% CI: 7 - NA months, 9 events; log-rank chi-square = 0.1, P = 0.8). **[C]** The mean expression of TGFB1 in the 10-patient $TGFB1^{high}$ subset was 5.38 ± 0.12 (median, range) = 5.32, 4.66 - 5.99). By comparison, the mean TGFB1 expression level for the 9-patient $TGFB1^{low}$ subset was 3.63 ± 0.24 (median, range = 3.85, 2.09 - 4.26).$TGFB1^{high}$ patients (median OS = 10 months, 95% CI: 8 - NA months, 9 events) exhibited a statistically insignificant worse OS than $TGFB1^{low}$ patients (median OS = 17 months, 95% CI: 7 - NA months, 9 events; log-rank chi-square = 0.5, P = 0.5).

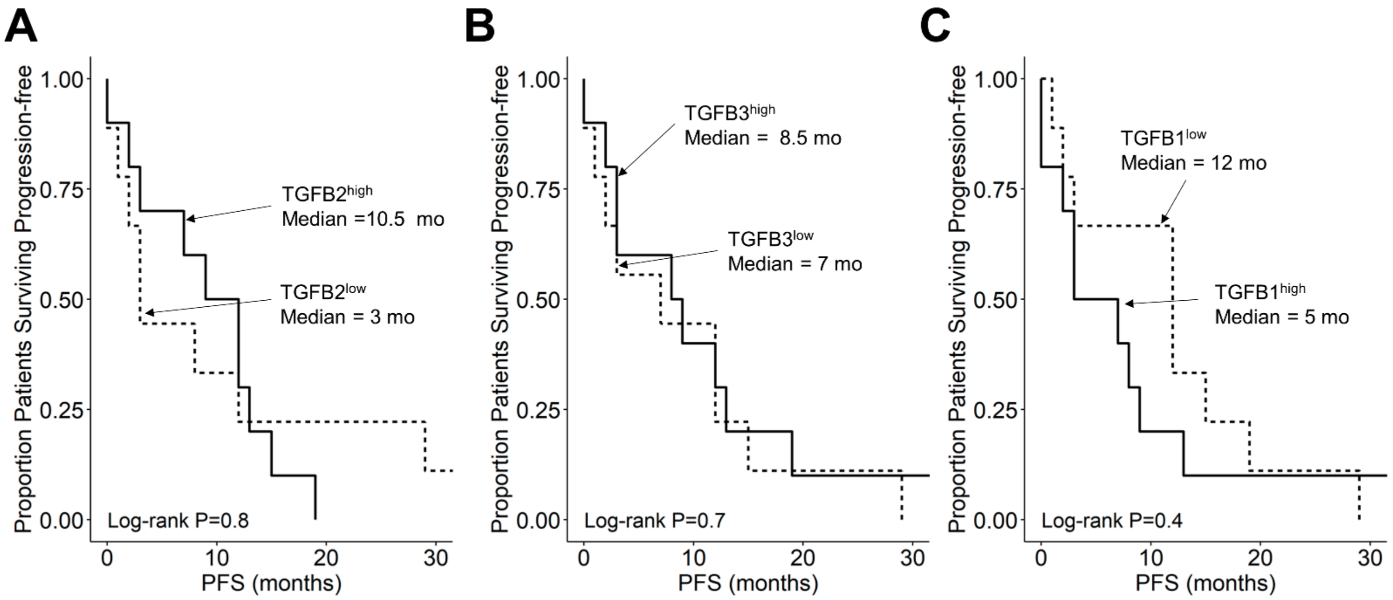

Figure S5. TGFB2 expression level does not affect PFS in DMG patients with tumor location outside pons/brainstem. Archived clinical outcome data for 19 H3K27M-mutant DMG patients, whose brain tumors were not localized to the brainstem/pons, obtained from cBioPortal for Cancer Genomics (https://pedcbioportal.kidsfirstdrc.org/) was combined with RNAseq-based mRNA expression data for TGFB2 **[A]**, TGFB3 **[B]**, and TGFB1 **[C]** to assess the potential impact of the respective TGFB isoform levels on PFS. For patients with missing information regarding progression of disease, death was used as the first event when evaluating the PFS outcome. Log_2-transformed, TPM-normalized RNAseq values were rank-ordered according to expression level for each TGFB isoform. Patients whose expression levels for a given TGFB isoform was greater than or equal to the median (solid line) were compared to the remaining patients (dashed line). [**A**] $TGFB2^{high}$ patients (median PFS = 10.5 months, 95% CI: 3 - NA months, 10 events) exhibited a statistically insignificant difference for PFS outcome when compared to $TGFB2^{low}$ (median PFS = 3 months, 95% CI: 2 - NA months, 9 events; log-rank chi-square = 0, P = 0.8). [**B**] $TGFB3^{high}$ patients (median PFS = 8.5 months, 95% CI: 3 - NA months, 10 events) exhibited a statistically insignificant difference for PFS outcome when compared to $TGFB3^{low}$ patients (median PFS = 7 months, 95% CI: 2 - NA months, 9 events; log-rank chi-square = 0.2, P = 0.7). **[C]** $TGFB1^{high}$ patients (median PFS = 5 months, 95% CI: 2 - NA months, 10 events) exhibited a statistically insignificant difference for PFS outcome when compared to $TGFB1^{low}$ patients (median PFS= 12 months, 95% CI: 3 - NA months, 9 events; log-rank chi-square = 0.6, P = 0.4).

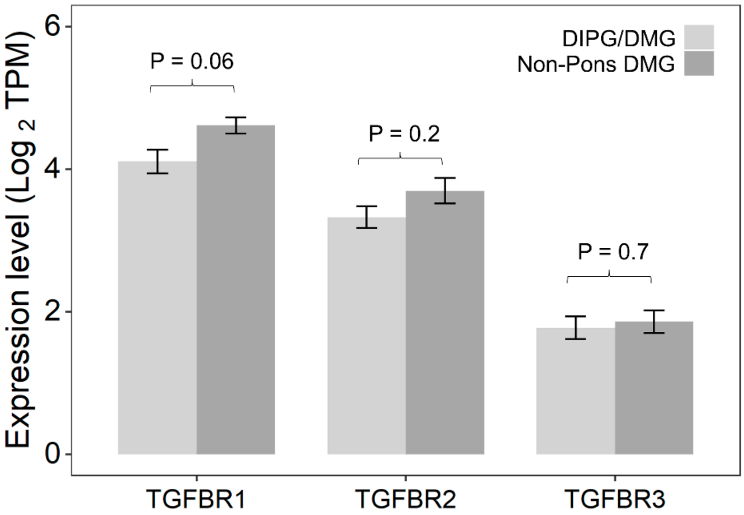

Figure S6. Expression of receptors for TGFB2 (TGFBR1/2/3) in Pons and Non-Pons DMG patients. Archived RNA-seq TGFBR1/2/3 expression data (https://pedcbioportal.kidsfirstdrc.org/) for 41 DIPG patients (light grey bars) including 11 DIPG patients with unknown H3K27M mutational status, 4 H3K27M-mutant DIPG patients and 26 H3K27M-mutant DMG patients, whose brain tumors were localized to the pons/brainstem, were compared to 19 H3K27M-mutant DMG patients, whose brain tumors were not localized to the brainstem/pons (dark grey bars, Non-Pons DMG). Statistical significance was assessed using $\log_2$-transformed, TPM-normalized expression values utilizing a two-way ANOVA model: fixed factors for receptor (TGFBR1, TGFBR2, TGFBR3) and patient subset (41 DIPG and 19 Non-Pons DMG patients) and one interaction term (receptor x patient subset). Contrasts were performed between DIPG and Non-Pons DMG samples for each of the receptor transcripts (reported FDR-adjusted P-values). None of the TGFB2 receptors exhibited a lower expression level in tumor samples from non-DIPG DMG patients to explain the observed lack of prognostic significance of higher TGFB2 levels in this patient group. DIPG patients exhibited a slightly reduced expression of TGFBR1 (mean = 4.1 ± 0.17; median = 4.18; range = 0.36 - 6.73) compared to Non-Pons DMG patients (mean = 4.61 ± 0.11; median = 4.71; range = 3.67 - 5.37) that was statistically borderline significant (linear contrast P = 0.06). TGFBR2 (mean = 3.32 ± 0.15, median = 3.48, range = 0.08 - 4.96 for DIPG patients; mean = 3.69 ± 0.18, median = 3.47, range = 2.58 - 5.38 for Non-Pons DMG patients; linear contrast P=0.2) and TGFBR3 (mean = 1.77 ± 0.16, median = 1.87, range = -1.64 - 4.09 for DIPG patients; mean = 1.86 ± 0.16, median = 1.83, range = 0.7 - 3.58 for Non-Pons DMG patients; linear contrast P=0.7) expression were not statistically different in DIPG and Non-Pons DMG patients.

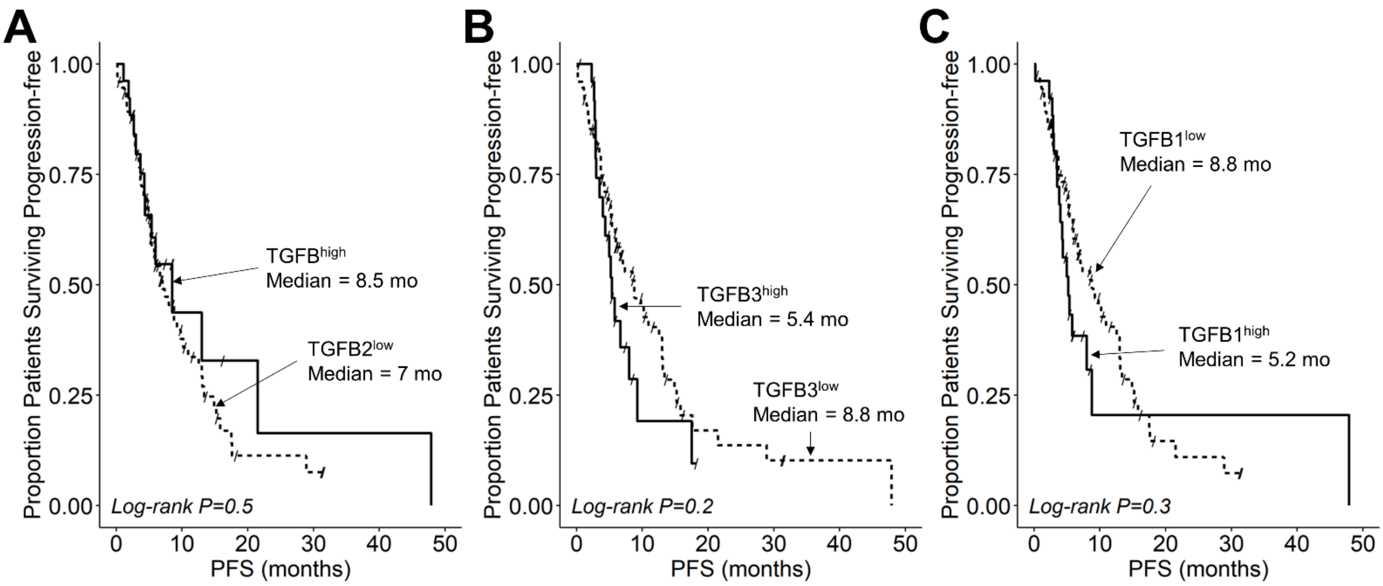

Figure S7. TGFB2 expression level does not affect PFS in pediatric GBM patients. Archived PFS data for 101 GBM patients obtained from cBioPortal for Cancer Genomics (https://pedcbioportal.kidsfirstdrc.org/) was combined with RNAseq-based mRNA expression data for TGFB2 **[A]**, TGFB3 **[B]**, and TGFB1 **[C]** to assess the potential impact of the respective TGFB isoform levels on PFS. Log_2-transformed, TPM-normalized RNAseq values were rank-ordered according to expression level for each TGFB isoform. Patients whose expression levels for a given TGFB isoform was greater than or equal to the upper quartile (solid line) were compared to the remaining patients (dashed line). **[A]** The mean expression level for TGFB2 in the 26-patient TGFB2^{high} subset was 12.56 ± 0.11 (median, range = 12.48, 11.92 - 13.62). By comparison, the mean expression level for TGFB2 in the 75-patient TBFB2^{low} subset was 10.46 ± 0.11 (median, range = 10.53, 8.17 - 11.92). Patients in the TGFB2^{high} and TGFB2^{low} subsets exhibited very similar PFS outcomes (median for TGFB2^{high}: 8.5 (95% CI: 4.4 - NA) months, 14 events; median for TGFB2^{low}: 7 (95% CI: 5.4 - 11) months, 53 events; log-rank chi-square = 0.6, P = 0.5). **[B]** The mean expression level for TGFB3 in the 26-patient TGFB3^{high} subset was 11.45 ± 0.11 (median, range = 11.42, 10.63 - 12.88). By comparison, the mean expression level for TGFB3 in the 75-patient TBFB3low subset was 9.38 ± 0.09 (median, range = 9.56, 7.14 - 10.58). Patients in the TGFB3^{high} and TGFB3^{low} subsets exhibited very similar PFS outcomes (median for TGFB3^{high}: 5.4 (95% CI: 4 - NA) months, 17 events; median for TGFB3^{low}: 8.8 (95% CI: 6 - 13) months, 50 events; log-rank chi-square = 1.6, P = 0.2). **[C]** The mean expression level for TGFB1 in the 26-patient TGFB1^{high} subset was 12.07 ± 0.08 (median, range = 11.91, 11.56 - 13.13). By comparison, the mean expression level for TGFB1 in the 75-patient TBFB1^{low} subset was 10.51 ± 0.1 (median, range = 10.71, 7.5 - 11.56). TGFB1^{high} and TGFB1^{low} subsets exhibited very similar PFS outcomes (median for TGFB1^{high}: 5.2 (95% CI: 4 - NA) months, 18 events; median for TGFB1^{low}: 8.8 (95% CI: 6 - 13) months, 49 events; log-rank chi-square = 1.1, P = 0.3).

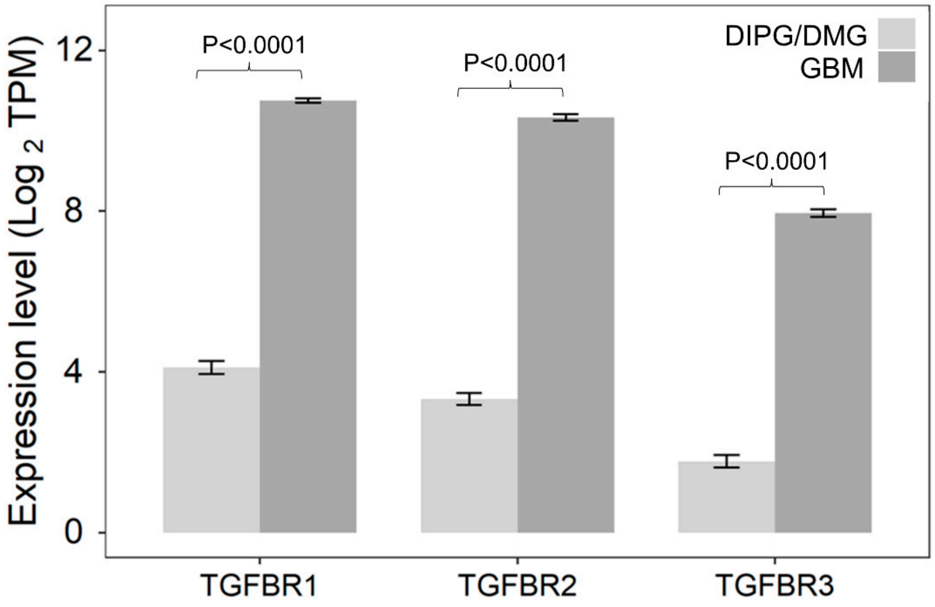

Figure S8. Expression of receptors for TGFB2 (TGFBR1/2/3) in DIPG and GBM patients.

Archived RNA-seq TGFBR1/2/3 expression data (https://pedcbioportal.kidsfirstdrc.org/) for 41 DIPG patients (light grey bars): including 11 DIPG patients with unknown H3K27M mutational status, 4 H3K27M-mutant DIPG patients and 26 H3K27M-mutant DMG patients, whose brain tumors were localized to the pons/brainstem, were compared to 116 pediatric GBM patients (dark grey bars). Statistical significance was assessed using log_2-transformed, TPM-normalized expression values utilizing a two-way ANOVA model: fixed factors for receptor (TGFBR1, TGFBR2, TGFBR3) and patient subset (41 DIPG and 116 GBM patients) and one interaction term (receptor x patient subset). Contrasts were performed between DIPG and GBM samples for each of the receptor transcripts (reported FDR-adjusted P-values). None of the TGFB2 receptors exhibited a lower expression level in tumor samples from GBM patients to explain the observed lack of prognostic significance of higher TGFB2 levels in this patient population. To the contrary, GBM patients exhibited significantly higher levels of all three receptors (linear contrast $P<0.0001$ for all comparisons) compared to DIPG patients (expression levels for DIPG patients reported in Figure S4). TGFBR1 exhibited 100-fold higher levels in GBM patients (mean = 10.75 ± 0.05; median = 10.73; range = 9.35 - 12.21). TGFBR2 exhibited a 128-fold increase in GBM patients (mean = 10.33 ± 0.08; median = 10.38; range = 8.35 - 12.7). TGFBR3 exhibited a 72-fold increase in GBM patients (mean = 7.95 ± 0.09; median = 7.91; range = 5.76 - 11.52).

Table S1. Increased TGFB2 mRNA expression levels in pediatric patients with DIPG or H3K27M-mutant GBM

Probeset	DIPG + pGBM with H3K27M (N=41) Mean ± SEM	Normal (N=2) Mean ± SEM	Fold Increase vs. Normal	Linear Contrast P-value
TGFB1_203084_at	5.1 ± 0	5.1 ± 0	0.97	0.9
TGFB1_203085_s_at	6 ± 0.1	7.1 ± 0.1	0.45	0.028
TGFB2_209908_s_at	4.1 ± 0.1	3.6 ± 0.1	1.40	0.4
TGFB2_209909_s_at	6.9 ± 0.2	5.6 ± 0.2	2.35	0.019
TGFB2_220406_at	4.7 ± 0	4.6 ± 0	1.08	0.8
TGFB2_220407_s_at	6.1 ± 0.2	4.9 ± 0.1	2.19	0.032
TGFB2_228121_at	9.7 ± 0.1	8.3 ± 0.1	2.71	0.006
TGFB3_1555540_at	4 ± 0	3.8 ± 0	1.10	0.8
TGFB3_209747_at	6.7 ± 0.1	7.9 ± 0.2	0.44	0.026

Table S2. TGFB2_220407_s_at mRNA probeset correlations with transcription factors in pediatric patients with DIPG or H3K27M-mutant GBM

Probeset	Correlation Coefficient vs. TGFB2_220407_s_at	P-value
TGFB2_220407_s_at	1	
TGFB2_209909_s_at	0.93	3.4×10^{-18}
TGFB2_228121_at	0.83	3.1×10^{-11}
ATF1_1558233_s_at	0.71	2.6×10^{-7}
ATF1_222103_at	0.67	1.4×10^{-6}
ATF1_1565269_s_at	0.61	2.2×10^{-5}
TGFB2_209908_s_at	0.6	3.9×10^{-5}
CREB1_204313_s_at	0.53	3.7×10^{-4}
CREB1_204314_s_at	0.53	3.5×10^{-4}
CREB1_204312_x_at	0.52	5.4×10^{-4}
ATF2_205446_s_at	0.51	6.3×10^{-4}
CREB1_214513_s_at	0.51	6.4×10^{-4}
FOXO3_231548_at	0.51	6.8×10^{-4}
CREB1_237289_at	0.45	3.1×10^{-3}
EP300_202221_s_at	0.42	6.7×10^{-3}
EP300_213579_s_at	0.41	7.2×10^{-3}
ATF2_212984_at	0.4	9.2×10^{-3}
TGFB2_220406_at	0.38	1.4×10^{-2}
USF1_231768_at	0.37	1.6×10^{-2}
CREB1_225572_at	0.35	2.4×10^{-2}
SP1_1553685_s_at	0.35	2.4×10^{-2}
POLR2A_217420_s_at	0.34	2.9×10^{-2}
SP1_224760_at	0.27	8.4×10^{-2}
TBP_203135_at	0.22	1.7×10^{-1}
FOXO3_217399_s_at	0.16	3.1×10^{-1}
FOXO3_210655_s_at	0.11	4.9×10^{-1}
FOXO3_224891_at	0.01	9.7×10^{-1}
FOXO3_204132_s_at	0	9.9×10^{-1}
SP1_214732_at	0	1.0×10^{0}
POLR2A_217415_at	-0.05	7.6×10^{-1}
USF2_215737_x_at	-0.07	6.7×10^{-1}
FOXO3_224889_at	-0.09	5.7×10^{-1}
SP1_224754_at	-0.12	4.5×10^{-1}
ATF2_1555146_at	-0.14	3.7×10^{-1}
HOXC10_218959_at	-0.16	3.2×10^{-1}
USF2_214879_x_at	-0.19	2.2×10^{-1}
CREB1_225565_at	-0.22	1.6×10^{-1}
POLR2A_202725_at	-0.24	1.4×10^{-1}
RFX1_226786_at	-0.36	2.0×10^{-2}

RFX1_206321_at	-0.38	1.6×10^{-2}
USF2_202152_x_at	-0.39	1.3×10^{-2}
FOXO3_204131_s_at	-0.43	5.3×10^{-3}
